# High order Interaction and Wavelet Convolution Network for visible infrared person reidentification

**DOI:** 10.1038/s41598-025-14978-x

**Published:** 2025-08-21

**Authors:** Li Ma, Rui Kong, XinGuan Dai

**Affiliations:** College of Communication and Information Engineering, Xi’an University of Science and Technology, Xi’an, 710054 China

**Keywords:** Visible-infrared person re-identification, High-order interaction, Wavelet convolution, Deep learning, Computer science, Information technology, Software

## Abstract

Visible-infrared person re-identification (VI-ReID) remains a challenging task due to significant cross-modal discrepancies and poor image quality. While existing methods predominantly employ deep and complex neural networks to extract shared cross-modal features, these approaches inevitably discard critical primitive features during high-level feature abstraction. To address this limitation, we propose the High-order Interaction and Wavelet Convolution Network (HIW-Net) that systematically integrates primitive features at multiple feature interaction stages, thereby compensating for information loss in High-order representations. Furthermore, our framework uses wavelet convolution to mine more diverse features and solve the problem of insufficient feature extraction. We create the RegDB_shape datasets with the help of the Segment Anything Model(SAM) tool to supplement the training set. Extensive experiments on the SYSU-MM01 and RegDB datasets show the superiority of the proposed HIW-Net over several other state-of-the-art methods, proves the effectiveness of this method.

## Introduction

Person Re-identification (Re-ID) is a computer vision task aimed at identifying the same individual by analyzing and comparing images captured at different times, from varying perspectives, and across different cameras. This technology is widely used in security monitoring, intelligent transportation, and smart retail^1-3^. Currently, methods such as QAConv-GS, AKA, SPT, FastReID, and CLIP-ReID have demonstrated strong performance in person re-identification within the visible light spectrum^4-8^. However, in practical scenarios, challenges such as poor lighting conditions, complex environments, weather variations, and day-night transitions hinder effective person re-identification based solely on visible light images. Infrared cameras, thermal images, and other devices can ignore these effects and image the human body. Therefore, many scholars are actively exploring cross-modal person re-identification between visible light and infrared modalities, proposing methods such as YYDS + CMKR, DEN, NFS, and FMCNet^9-12^, which aim to develop invariant feature representations across modalities and camera perspectives.

**Fig. 1:**
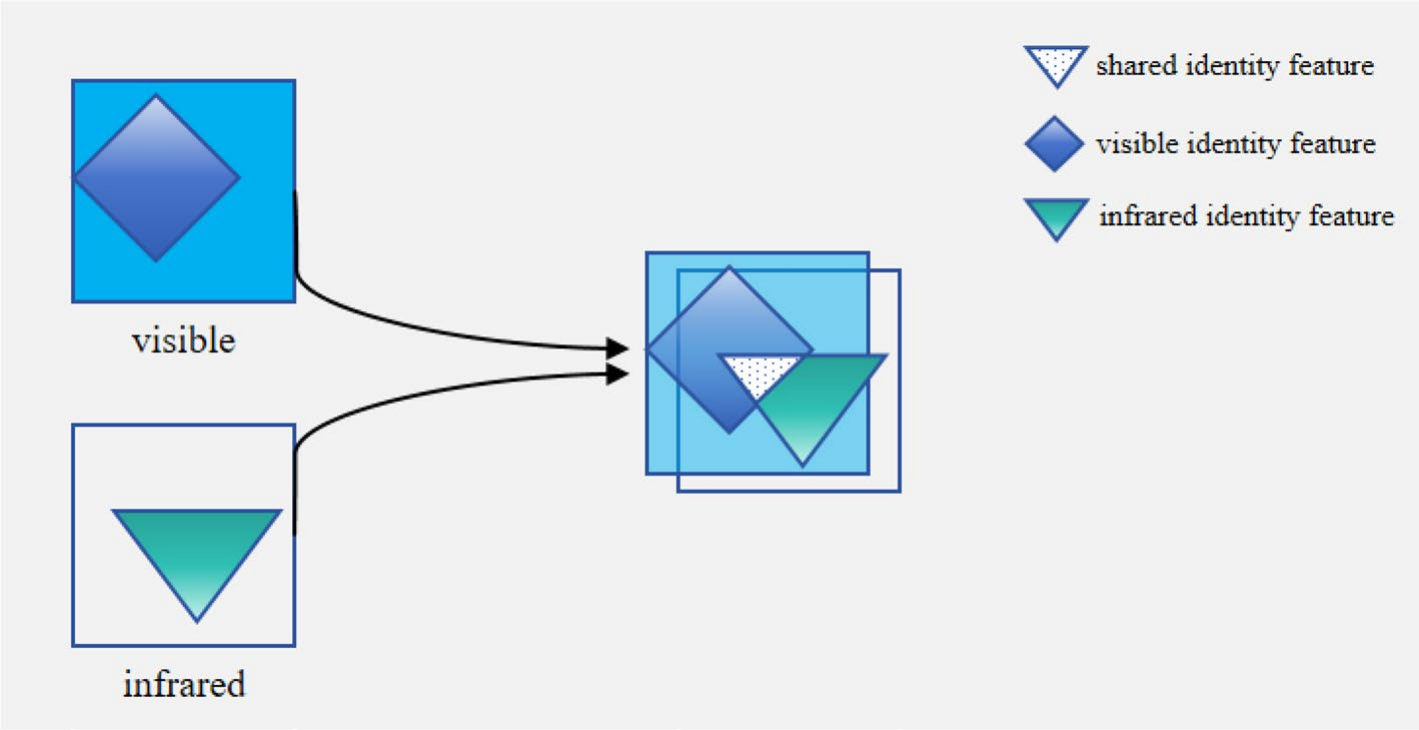
Visualization of modal differences. The diamonds represent the identity feature representations in the visible light modality, and the triangles represent the identity feature representations in the infrared modality. In the overlap of the schematics for both modalities, the white triangle region indicates shared invariant features of the same person across different modalities, while the non-overlapping regions show the differences in identity feature representations between the visible light and infrared modalities.

The difference between visible light and infrared images represents a significant challenge in this domain. Visible light images primarily rely on visible light reflected by objects, while infrared images depend on thermal radiation emitted by objects, resulting in substantial differences between the two modalities in color, texture, and contrast^13-15^. As shown in Fig. 1, while visible and infrared images contain identity features of the same person, the identity features of the same person in the two modalities are different, but also have same parts. (represented by the white triangle region in Fig. 1, referred to as shared features in this paper). The main challenge is to learn these features effectively. We divide existing methods into two categories, nongenerative-based and generative-based.

Generative methods such as the GECNet network, GECNet uses grayscale transformation to make visible and infrared images similar^16^. Some methods employ GANs for modal transformation, converting images from one modality into another to minimize modal differences^17-19^. In this field, VIS-IR image pairs are often essential for guiding modality generation, but some mainstream datasets, such as the SYSU-MM01 dataset, do not fulfill this requirement. The absence of VIS-IR image pairs can introduce noise into the generated images. Additionally, generative methods have notable limitations in practical applications.

Non-generative approaches embed features from different modalities into a unified feature space^11,20,21^. However, this process may discard certain features from visible and infrared modalities, including critical details essential for person re-identification. Most existing methods focus on reducing modal differences, such as bidirectional modality information interaction network using of Dynamic Aggregation (DA) module^54^ and modality shared-specific features cooperative separation network^42^, which are represented in Fig. 1 as increasing overlapping white triangular areas. Although these approaches achieve promising results, they ignore feature loss at deeper network stages and fail to fully utilize shared features.

**Fig. 2:**
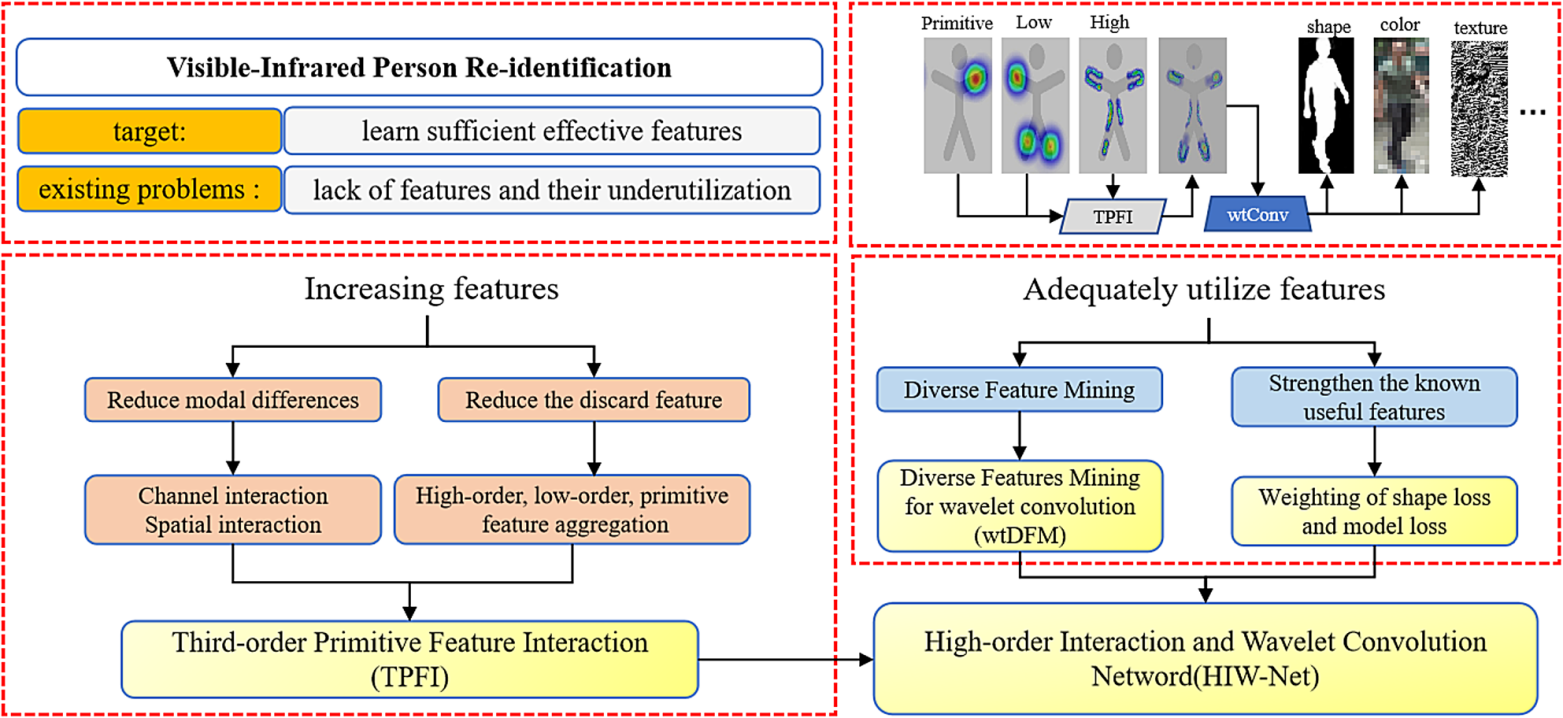
HIW-Net module function diagram.

The HIW network proposed in this paper not only increases shared features but also focuses on their effective utilization. It consists of a third-order primitive feature interaction module (TPFI) and a diversified feature mining module based on wavelet convolution (wtDFM). Firstly, to reduce the differences between different modalities, the TPFI module is used to interact the features of different modalities through channel and spatial dimensions. At the same time, to reduce the potential loss of features during the network extraction process, the primitive features, low order features, and High-order features are aggregated separately in the channel and spatial dimensions. Low order and original features are used to compensate for High-order features, ensuring sufficient extraction of shared features, and reducing the redundant parameter amount of aggregation in the entire stage. Then, the wtDFE module applies wavelet convolution branches with different receptive fields to the features after TPFI. This enhances the utilization of shared features and explores diversified features, including shape, color, texture, and more abstract attributes(as shown in Fig. 2).

As shown in Fig. 2, to achieve visible-infrared person re-identification and address the issues of insufficient feature extraction and underutilization of features in existing methods, the proposed HIW network introduces two novel modules: TPFI and wtDFM. In addition, inspired by the SGIEL^22^, where shape, as an invariant shared feature across modalities, is the key information to achieve person re-recognition. SGIEL uses orthogonal projections to eliminate shape information in one projection, which is used to force the network to learn features other than shape. In this paper, the opposite idea is taken, where only shape information is retained and used to force the network to enhance the learning of shape features. In the first stage, the network inputs images that contain all the information, and in the second stage the network inputs images that contain only shape information, by weighing the shape loss from the second stage with the loss from the first stage, and fed back into the training process, the network is trained to better utilize shape features.

**Fig. 3:**
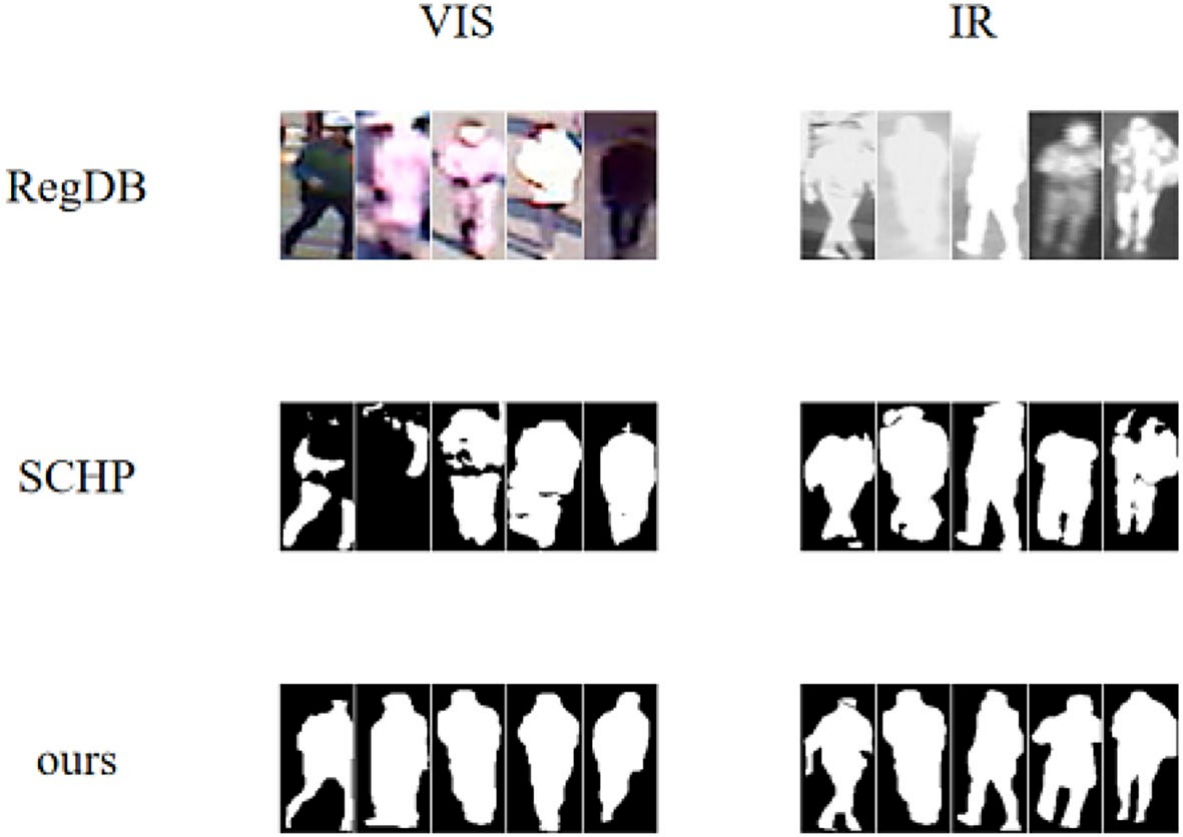
Comparison of shape diagrams from the RegDB_shape dataset generated by SCHP and manually annotated diagrams from this paper.

In addition, the low quality of the RegDB datasets hinders the effective creation of shape diagrams using existing human body analysis networks^23,25^. To address this, this paper uses SAM to annotate the datasets and construct the RegDB_shape datasets^26^. As shown in Fig. 3, the newly created datasets addresses issues of incomplete person profiles and offers valuable support for research in person re-identification.

The main contributions are as follows:

1. This paper proposes a novel third-order primitive feature interaction module (TPFI) that minimizes inter-modal differences through channel and spatial dimension interactions while mitigating shared feature loss through third-order feature aggregation.
2. The Wavelet convolution was introduced to achieve diversified feature mining. The proposed wtDFM module utilizes different wavelet convolution branches to fully exploit shared features.
3. The HIW network integrates the strengths of TPFI and wtDFM to enhance cross-modal shared feature representation and maximize the utilization of shared features. In addition, shape loss and modality loss were weighed to optimize the total loss and enhance the utilization of shape features.
4. This paper creates RegDB_shape datasets to advance research in the field of person re-identification.
5. Extensive experiments demonstrate that the HIW network outperforms other networks on the mainstream SYSU-MM01 and RegDB datasets.

## Related work

Current visible-infrared person re-identification (VI-ReID) methods can be categorized into generative and non-generative approaches^55^. Generative models primarily employ generative adversarial networks (GAN) or encoder-decoder modules to facilitate mutual conversion between the two modalities or to create intermediate ones. Sometimes, they also require the fusion of visible and infrared images, such as the SIHP network^30^, which uses corresponding visible and infrared images to explore complex interactions in image fusion. However, generative methods need VIS-IR image pairs, which have certain limitations in practical applications. Therefore, this paper mainly focuses on non-generative methods.

This paper focuses on enhancing the baseline AGW method for cross-modal person re-identification. The AGW method incorporates a Non-local Attention module into ResNet50, primarily utilizing a $1 \times 1$ convolution kernel. This module enables the model to capture long-range dependencies in images, compute the weighted sum of all positional features, and enhance the feature representation capability. Generalized-mean Pooling (GeMPooling), a learnable pooling layer, is used to provide a continuous transition between maximum pooling and average pooling, GeMPooling adjusts its pooling behavior via a learnable hyperparameter ${p_k}$, it behaves similarly to maximum pooling as ${p_k}$ approaches infinity and is equivalent to average pooling when ${p_k}=1$. Specifically, GeMPooling calculates the generalized average of each feature channel, adjusting ${p_k}$ enhances the contrast of the pooled feature map, emphasizing more significant image features. AGW puts Weighted Regression Triplet loss, an improvement over traditional triplet loss, this loss function inherits the advantages of relative distance optimization between positive and negative pairs while avoiding the introduction of additional margin parameters, it optimizes triplet positive set *P* and negative set *N* in batches using a weighted regularization approach, thereby improving the model’s discrimination ability[28]. But, AGW also has many drawbacks, it merely concatenates visible and infrared features, resulting in large modal differences. There is also no diversity mining of the features, which are directly input into the triplet loss function, leading to underutilization of the features.

To obtain more effective features, this paper aggregates features learned at different stages, this strategy validated by the ANN network proposed by Zhu Z^29^. The ANN network puts Asymmetric Fusion Non-local Block (APNB) module, which integrates features across different levels while accounting for long-range dependencies. This approach significantly enhances performance, demonstrating the effectiveness of feature aggregation at various levels in tasks such as semantic segmentation and classification. However, full-stage feature aggregation may lead to feature redundancy and high computational costs.

To reduce modality differences, the features extracted from visible light and infrared images can be interacted with in channel and spatial dimensions, such as the SCSN network uses an Residual Dual Attention Module (RDAM) module composed of Channel-wise Attention Module (CAM) and Residual Spatial Attention Module (RSAM). In addition, The SCSN network mines diverse salient features through the use of cascading Salient Feature Extraction (SFE) units. SFE suppresses salient features learned in the previous cascading stage, adaptively extracts additional potential salient features, and integrates these features into the final representation through cascading. However, a person’s unique features are finite, and excessive suppression may lead the network to learn non-robust features, resulting in feature vector redundancy and a dilution of salient features^28^.

After extracting sufficient features, it is also necessary to make full use of these features and strengthen the diversity mining of features. The SGIEL forcibly removes body shape-related features, compelling the VI-ReID model to extract additional features shared across modalities for recognition. By orthogonal decomposition of the feature space, shape-related features are captured in a subspace, while shape-erased features are mapped to an orthogonal complement space, enhancing feature diversity^22^. However, this method relies heavily on prior knowledge of body shape, making it susceptible to inaccuracies or incompleteness in body shape information, which can adversely impact feature extraction.

The DEEN Network^32^ uses different expansion rates to simulate different receptive fields and extract multi-scale features, but this may result in tiny details being overlooked. Dilated convolution can introduce more gaps, leading to information loss and ambiguity. Although dilated convolution expands receptive fields, it significantly increases computational cost. As the expansion rate increases, the interval between elements in the convolution kernel becomes larger, which means that each element can cover a larger input area, which increases the computational complexity and thus affects the utilization of GPU.

Inspired by the above methods, between the full-stage aggregation of the ANN method and the second-order aggregation of the SCSN method, this paper proposes the third-order primitive feature interaction, which takes ResNet50 as the backbone, take the features of the image after a simple convolution as the primitive features. From the third stage onwards, these primitive features are inputted into channel-wise and spatial feature interactions. This method, termed third-order primitive feature interaction (TPFI), compensates for potential losses of shared features during deep network extraction. At the cost of increasing a small amount of computational cost, it significantly reduces the loss of shared features in the deep network extraction process.

To enhance the diversity of shared features, this paper uses Wavelet Transform (WT) to create a wavelet convolution module, it achieves a large receptive field without excessive parameterization^33^. Wavelet convolution can better capture the low-frequency information in the image, thus enhancing the response to shape, which is well combined with the idea of using shape loss and modality loss to get the total loss to enhance the network’s extraction of shape features, and further enhance the network’s response to shape features.

## Method

**Fig. 4:**
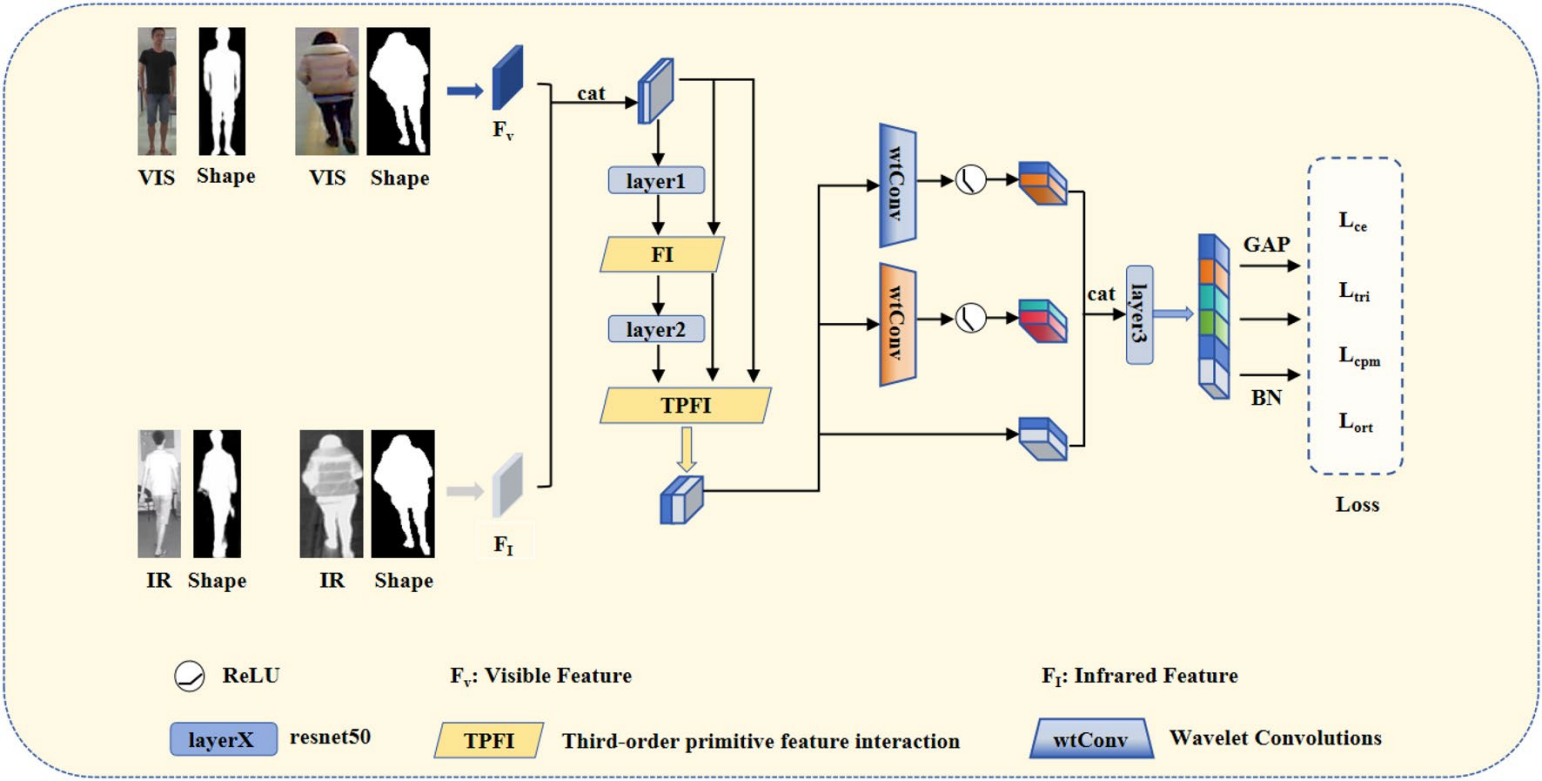
The structure of the HIW-Net. The FI module performs interactions across channel and spatial dimensions, aggregating higher-order and low-order features to reduce modality differences. and feature loss. TPFI, based on FI, aggregating primitive features (F_v_ and the features after F_I_ concatenation), decreasing feature loss in deep networks. The wtConv module extracts image features of different frequencies from a frequency perspective. Total loss is the weighted sum of Shape Loss and Modality Loss. Both losses are the sum of ${L_{ce}}$, ${L_{tri}}$, ${L_{ort}}$ and ${L_{cpm}}$. Shape Loss uses processed shape images of visible and infrared images, while Modality Loss uses visible and infrared images directly.

### Model architecture

Figure 4 outlines the network framework for High-order Interaction and Wavelet Convolution Network for visible-infrared person re-identification (HIW-Net). It uses Resnet50 as the backbone and consists of two passes.

In the first pass, visible and infrared images are input. The preprocessing stage of Resnet50 extracts primitive features. Then, for each layerX, it aggregates the input (low-level features), output (high-level features), and primitive features. In the first stage, as the layer’s input is the primitive feature, it uses basic feature interaction (FI). For other stages, it uses third-order primitive feature interaction (TPFI), which involves interaction in both channel and spatial dimensions. For the RegDB datasets, only the first two stages of Resnet50 are used, and for the SYSU datasets, the first three stages are used. Then, The wavelet convolution module splits the aggregated features into diverse frequency bands. It then processes each band with convolutions, using branches with different wavelet convolution kernel sizes to capture more varied feature information. Finally, the model loss, which is the sum of ${L_{ce}}$, ${L_{tri}}$, ${L_{ort}}$ and ${L_{cpm}}$, is calculated using these diverse features.

The second pass takes the shape images of visible and infrared images (containing only human shape features) as input. The calculation method is the same as the first pass, yielding the shape loss.

The total loss is the weighted sum of model loss and shape loss.

### Third-order primitive feature interaction

Figure 4 illustrates the structure of the third-order primitive feature interaction module. In this paper, features preceding the layer are called low-order feature ${f_l}$, features following the layer are called High-order feature ${f_h}$, and features resulting from the initial image convolution, batch normalization(BN), ReLU, and max-pooling operations are defined as primitive feature

** Channel interaction**: Channel interaction between the primitive feature ${f_p}$ and the higher-order feature ${f_h}$, use three convolution $\varphi _{g}^{p}$, $\varphi _{t}^{p}$, $\varphi _{v}^{p}$ to preprocess the primitive and higher-order features, generating three compact outputs $\varphi _{g}^{p}\left( {{f_p}} \right)$, $\varphi _{t}^{p}\left( {{f_h}} \right)$, $\varphi _{v}^{p}\left( {{f_p}} \right)$with the same feature dimensions as the primitive feature, the feature map is flattened in the last dimension, and then use matrix multiplication and softmax to calculate the channel similarity of ${f_p}$ and ${f_h}$, then obtain the channel similarity matrix.

1 $$M_{C}^{p}({C_p} \times {C_p})=soft\hbox{max} (\varphi _{g}^{p}({f_p}) \times \varphi _{t}^{p}({f_h})),$$

Then, the preprocessed High-order feature $\varphi _{v}^{p}\left( {{f_p}} \right)$and channel similarity matrix $M_{C}^{p}$ are multiplied to achieve feature interaction across different stages. Then use a $1 \times 1$ convolution $\varphi _{w}^{{ph}}$ to restore the interaction features to same shape as the higher-order feature ${f_h}$:

2 $$f_{p}^{h}=\varphi _{w}^{{ph}}(M_{C}^{p} \times \varphi _{v}^{p}({f_p})),$$

Use the same method to perform channel interaction on low-order features ${f_l}$ and High-order features ${f_h}$ to obtain:

3 $$f_{l}^{h}=\varphi _{w}^{{lh}}(M_{C}^{l} \times \varphi _{v}^{l}({f_l})),$$

Finally, we utilize matrix addition to perform feature aggregation along the channel dimension in the third-order primitive feature interaction module:

4 $$f_h^C = {f_h} + f_l^h + f_p^h$$

**Spatial interaction:** Then, we performed the spatial interaction of High-order features of channel aggregation have been completed $f_{h}^{C}$, low-order features ${f_l}$ and primitive features ${f_p}$,this operation is similar to channel interaction, the difference is that in the feature pre-processing stage, file the channel dimension with the features of the spatial dimension, so that the spatial size of the pre-processed low-order features ${f_l}$ and primitive features ${f_p}$ is consistent with the High-order. Finally, we obtain the output of the third-order primitive feature interaction.

5 $${f_{TPFI}}=f_{h}^{C}+\psi _{w}^{{lh}}(M_{S}^{l} \times \psi _{p}^{l}({f_l}))+\psi _{w}^{{ph}}(M_{S}^{p} \times \psi _{v}^{p}({f_p})),$$

where, $\psi$denotes $1 \times 1$ convolution in spatial interaction, and ${M_S}$ represents the spatial similarity matrix.

### Diverse features mining for wavelet Convolution

**Parameter efficiency and Multi-Frequency emphasis:** For *l*-level decomposition and a ${\text{k}} \times {\text{k}}$ kernel, wtConv’s[34] parameters scale as $\left( {l \cdot 4 \cdot c \cdot {k^2}} \right)$, whereas its effective receptive field(ERF) grows as $\left( {{2^l} \cdot k} \right)$. For example, a 3-level wtConv with $3 \times 3$ kernels achieves a $24 \times 24$ ERF using only 117 parameters(108 parameters plus bias terms) per channel, compared to 576 parameters for a $24 \times 24$standard convolution.

By focusing convolutions on low-frequency subbands, wtConv enhances shape bias and robustness to high-frequency noise. This aligns with the observation that low frequencies encode structural information, while high frequencies correspond to textures.

**Wavelet transform integration with CNNs:** The wtConv serves as a drop-in replacement for depth-wise convolutions in existing architectures. Its implementation requires no architectural modifications, ensuring compatibility with standard training pipelines and downstream tasks.


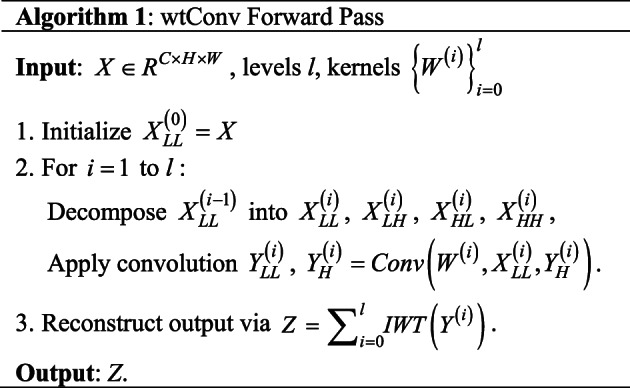


This paper uses $3 \times 3$ and $5 \times 5$ wtConv branches to extract features. The network concatenates features from different branches to obtain the final feature:

6 $${f^*} = {\theta _{1 \times 1}}({F_{\operatorname{Re} LU}}(\phi _{wtConv}^3({f_{TPFI}}) + \phi _{wtConv}^5({f_{TPFI}}) + {f_{TPFI}}))$$

where, ${\theta _{1 \times 1}}$represents a convolution of kernel size 1, changing dimension as same as ${f_{TPFI}}$.

### Loss functions

This section describes several loss functions for network training. It introduces shape images, performs two similar computations, and calculates the total loss via a simple weighted sum.

**Identity classification loss** ${L_{id}}$ uses the cross - entropy loss function^51^. This helps the model learn to distinguish pedestrians’ identity features and increases inter - class differences. It classifies the output feature ${f^*}$ to mitigate the negative impact of modality differences, ensuring the network correctly identifies different identities.

7 $${L_{ce}} = - \frac{1}{N}\sum\nolimits_{i = 1}^N {\sum\nolimits_{c = 1}^C {{y_{i,c}}\log ({p_{i,c}})} }$$

where, *N* represents the number of samples, *C* the number of classes, ${y_{i,c}}$ is the one - hot encoded labels, and ${p_{i,c}}$ is the predicted probabilities.

**Triplet loss** To learn a cross - modal shared metric space, this paper adopts triplet loss^49^. It narrows anchor - positive pairs (same - identity cross - modal samples) and widens negative pairs (different - identity samples), enhancing the feature space’s discriminative power.

8 $${L_{tri}} = \frac{1}{N}\sum\nolimits_{i = 1}^N {\max (d({a_i},{p_i}) - d({a_i},{n_i}) + \alpha ,0)}$$

where, $d\left( \cdot \right)$ denotes the Euclidean distance metric, ${a}$ is the margin hyperparameter, ${a_i}$ represents the anchor sample, ${p_i}$ the positive sample, and ${n_i}$ the negative sample.

**Orthogonality loss** In order to ensure the diversity of features, this paper uses orthogonal loss^50^ to constrain the orthogonality between feature vectors of different branches and reduce feature redundancy. Orthogonal loss is represented by:

9 $${L_{ort}} = \sum\limits_{m = 1}^{i - 1} {\sum\limits_{n = m + 1}^i {(f_ + ^{mT}f_ + ^n)} }$$

where, *m* and *n* are the *m-th* and *n-th* features generated from ${f_{TPFI}}$, respectively.

**Center-Guided Pair Mining Loss.** The center-guided pair mining loss, proposed by Zhang Y et al.^32^, is designed for generating diversified features for multi-branch network structures. It narrows intra-class distances across modalities, reducing VIS-IR modality gaps, while widening distances between generated and original features to mine diverse cross-modal clues. Crucially, it ensures inter-class distances exceed intra-class ones.

10 $$L({f_v},{f_n},f_{{v+}}^{i})={[D(f_{n}^{j},f_{{v+}}^{{i,j}}) - D(f_{v}^{j},f_{{v+}}^{{i,j}}) - D(f_{v}^{j},f_{v}^{k})]_+},$$

where, ${f_v}$ and ${f_n}$ are the VIS and IR features from TPFI block, $f_{{v+}}^{i}$ is the features generated from the *i-th* branch of the ${f_v}$. $D\left( { \cdot , \cdot } \right)$ is the Euclidean distance between two features. *j*, *k* are different identities in a mini-batch, ${\left[ x \right]_+}=max\left( {x,0} \right)$.

In Eq. (10), $f_{n}^{j}$ represents the infrared feature of identity *j*, $f_{v}^{j}$ represents the visible feature of identity *j*, and $f_{{v+}}^{{i,j}}$ represents the feature of generated by $f_{v}^{j}$ in the *i* branch. The first term reduces the distance between newly - generated visible features and original infrared ones, thus decreasing the modality difference. The second ${L_{ort}}=\sum\limits_{{m=1}}^{{i - 1}} {\sum\limits_{{n=m+1}}^{i} {(f_{+}^{{mT}}f_{+}^{n})} } ,$term increases the distance between newly - generated visible features and original visible ones, prompting the network to learn diverse features. The third term ensures the intra - class distance is smaller than the inter - class distance.

Similarly, for the feature generated by ${f_n}$ in branch *i*, the loss function that needs to be satisfied is:

11 $$L({f_v},{f_n},f_{{n+}}^{i})={[D(f_{v}^{j},f_{{n+}}^{{i,j}}) - D(f_{n}^{j},f_{{n+}}^{{i,j}}) - D(f_{n}^{j},f_{n}^{k})]_+},$$

Therefore, the final CPM loss can be expressed as:

12 $${L_{cpm}}=L({f_v},{f_n},f_{{v+}}^{i})+L({f_v},{f_n},f_{{n+}}^{i})$$

**Total Loss.** This paper employs four loss functions. The network undergoes two computational passes. In the first pass, visible and infrared images are input, and the network computes the sum of the four loss functions to obtain the modality loss.

13 $${L_{ml}}=L_{{_{{id}}}}^{m}+L_{{_{{tri}}}}^{m}+L_{{_{{ort}}}}^{m}+L_{{_{{cpm}}}}^{m},$$

where, $L_{*}^{m}$ represents the loss calculated using modal images.

In the second pass, the network takes shape images, derived from visible and infrared images, as input. It calculates the sum of the four loss functions to obtain the shape loss.

14 $${L_{sl}}=L_{{_{{id}}}}^{s}+L_{{_{{tri}}}}^{s}+L_{{_{{ort}}}}^{s}+L_{{_{{cpm}}}}^{s},$$

where, $L_{*}^{S}$ represents the loss calculated using shape images.

Finally, the network is trained by minimizing the sum of modal and shape losses.

15 $${L_{total}}=\lambda {L_{ml}}+(1 - \lambda ){L_{sl}}$$

where, $\lambda$ denotes the weight that adjusts the loss ratio to control the proportion of shape enhanced features in the network. Experimental exploration shows the network performs best when $\lambda$ = 0.9.

## Experiments

This paper conducted experiments on two public datasets, SYSU-MM01 and RegDB, to evaluate the effectiveness of the proposed method and compare it with recent approaches, demonstrating its superiority. Additionally, ablation experiments were conducted to assess the contributions of TPFI, wtDFM, and ShapeLoss.

### Dataset and evaluation protocol

The RegDB dataset contains a total of 412 people, each person has 10 visible light images and corresponding 10 thermal images, these images exhibit variations in body posture, capture distance, and lighting conditions, among the 412 people, there are 254 females and 158 males. In addition, 156 people were photographed from the front and the remaining 256 people were photographed from other angles. The images in this dataset are small and have poor clarity. The RGB and thermal images for each identity are in a one-to-one correspondence^34^. The SYSU-MM01 dataset contains images of 491 people captured by 4 RGB cameras and 2 infrared cameras, totaling 30,071 RGB images and 15,792 infrared images. For testing, it supports two evaluation settings: all-search mode and indoor-search mode. The query set contains 3,803 images captured from IR cameras 3 and 6 in both settings, while the gallery set in all-search mode includes all visible images from the four RGB cameras. In indoor-search mode, the gallery set includes images only from the two indoor RGB cameras.

Shape dataset. The dataset is converted into shape maps. The SCHP network is used to create shape maps for the SYSU-MM01 dataset^23^. However, due to the low resolution and poor image quality of the RegDB dataset, existing human body analysis networks produce unsatisfactory results^24,25^. This paper manually annotates the RegDB dataset to create the first high-quality RegDB shape dataset.

To evaluate the performance of the network, this paper uses two evaluation metrics: Rank and Mean Average Precision (mAP). The Rank indicator, particularly Rank-1, is a key metric for evaluating the performance of person re-identification algorithms. It indicates the proportion of correctly identified samples ranked first by the algorithm. A higher Rank-1 value signifies better performance in person re-identification tasks^35^. The mAP is another widely used evaluation metric. It measures the model’s average performance across all categories and is calculated by averaging the Average Precision(AP) of each query. mAP accounts for both precision and recall during the query process, providing a more comprehensive assessment of person re-identification performance^36^.

### Implementation details

This paper uses a single RTX 4090 GPU for experiments. After preprocessing the input image using horizontal flipping, random cropping, and random erasing techniques, the preprocessed image is fed into the network, which uses ResNet50 as its backbone. For the RegDB datasets, the fourth stage of ResNet50 is removed, the TPFI module proposed in this paper is added to the first and second stages, and the wtDFM module is added to the second stage. For the SYSU-MM01 datasets, the TPFI module is added after each of the first three stages, and the wtDFM module is added after the third stage. In the first 10 rounds of warming up the model, the learning rate increases from 0.01 to 0.1, then remains unchanged at 0.1 from 10 to 20 rounds. It then decreases to 0.01 from 20 to 80 rounds and to 0.001 from 80 to 120 rounds. Beyond 120 rounds, the learning rate is further reduced to 0.0001, for a total of 150 training rounds. The network uses the ImageNet pre-trained weight file.

### Comparison with state-of-the-art methods

This paper compared the proposed method with existing state-of-the-art VI-ReID methods, including FMCNet^12^, SGIEL^22^, DEEN^32^, PMT^37^, AGMNet^38^, LCNL^39^, MCJA^40^, DCPLNet^41^, MPMN^42^, PMCM^43^, CSDN^44^, MIP^45^, AGCC^46^, RCC^47^, DNS^48^, MSCMNet^52^, LAReViT^53^. Extensive experiments demonstrate that the HIW-Net proposed in this paper achieves superior or comparable performance on both the SYSU-MM01 and RegDB datasets.

**RegDB.** As shown in Table 1, for the VIS-to-IR mode of RegDB, the HIW-Net achieves a Rank-1 accuracy of 94.88%, representing the best performance, while the mAP metric demonstrates comparable performance. For the IR to VIS mode, a Rank-1 accuracy of 94.32% is achieved, representing the best performance, while mAP achieves the second-best result at 87.18%.

**SYSU-MM01.** As shown in Table 2, in the all-search mode of SYSU-MM01, the HIW-Net achieves the best performance in Rank-1, Rank-10, and Rank-20, achieving 85.05%, 97.67%, and 99.67%, respectively, which are 8.23%, 0.07%, and 0.36% higher than the second-best results. For the indoor-search mode, achieves the best performance in Rank-1, Rank-10, and mAP, achieving 87.50%, 99.32% and 87.75%, surpassing the second-best result by 3.29%, 0.32% and 0.92%. In other metrics, it demonstrated performance comparable to the best results.

The triplet loss employed by HIW-Net emphasizes the distance relationships between difficult sample pairs, aiming to place the ranking of target people images at front and enhance the Rank index. While the network effectively captures the shape and other key features of target people, it does not sufficiently distinguish between non-target people. This lack of fine-grained discrimination causes non-target people to appear relatively higher in the overall ranking, thereby affecting the mAP.

**Table 1:** Comparison of the proposed method with state-of-the-art approaches on the RegDB dataset. Bold values indicate the best performance, while underlined values represent the second-best results.

methods	venue	VIS to IR		IR to VIS	
		rank-1	mAP	rank-1	mAP
FMCNet^[[38]]^	CVPR22	89.12	84.43	88.38	83.86
SGIEL^[[23]]^	CVPR23	91.07	85.23	92.18	86.59
DEEN^[[33]]^	CVPR23	91.1	85.1	89.5	83.4
PMT^[[39]]^	AAAI23	84.83	76.55	84.16	75.13
AGMNet^[[40]]^	IEEE23	88.40	81.45	85.34	81.19
LCNL^[[41]]^	IJCV24	85.6	78.7	84.0	76.9
MCJA^[[42]]^	IEEE24	91.80	86.08	88.06	83.06
DCPLNet^[[43]]^	TIT24	*94.2*	87.3	91.7	84.8
MPMN^[[44]]^	TMM24	85.3	76.4	83.8	75.2
PMCM^[[45]]^	2024	93.09	**89.57**	91.44	87.15
CSDN^[[46]]^	2024	89.0	84.7	88.2	82.8
MIP^[[47]]^	2024	91.26	85.90	92.38	85.99
AGCC^[[48]]^	2024	92.59	86.18	91.35	84.92
RCC^[[49]]^	2024	92.00	88.01	90.01	86.15
DNS^[[50]]^	ECCV24	93.48	*88.10*	*93.01*	**88.56**
MSCMNet^[[54]]^	2025	90.4	81.2	87.7	78.2
LAReViT^[[55]]^	2025	90.4	84.7	90.5	85.3
HIW(ours)		**94.88**	86.59	**94.32**	*87.18*

**Table 2:** Comparison of the proposed method with state-of-the-art approaches on the SYSU-MM01 dataset. Bold values indicate the best performance, while underlined values represent the second-best results.

methods	Venue	All-Search				Indoor-Search			
		rank-1	rank-10	rank-20	mAP	rank-1	rank-10	rank-20	mAP
FMCNet^[[38]]^	CVPR22	66.34	-	-	62.51	68.15	-	-	74.09
SGIEL^[[23]]^	CVPR23	75.18	96.87	99.13	70.12	78.40	97.46	98.91	81.20
DEEN^[[33]]^	CVPR23	74.7	*97.6*	99.2	71.8	80.3	99.0	99.8	83.3
PMT^[[39]]^	AAAI23	67.53	95.36	98.64	64.98	71.66	96.73	99.25	76.52
AGMNet^[[40]]^	IEEE23	69.63	96.27	98.82	66.11	74.68	97.51	99.14	78.30
LCNL^[[41]]^	IJCV24	70.2	96.4	99.0	68.0	76.2	98.2	99.8	80.3
MCJA^[[42]]^	IEEE24	74.48	96.99	*99.31*	71.34	82.79	98.88	**99.92**	85.26
DCPLNet^[[43]]^	TIT24	74.0	96.5	98.9	70.3	78.3	98.7	*99.8*	81.9
MPMN^[[44]]^	TMM24	70.6	96.2	98.7	67.5	75.9	98.1	99.6	80.2
PMCM^[[45]]^	2024	75.54	97.49	99.30	71.16	81.52	98.99	99.71	84.33
CSDN^[[46]]^	2024	75.2	96.6	98.8	71.8	82.0	98.7	99.5	85.0
MIP^[[47]]^	2024	70.84	-	-	66.41	78.80	-	-	79.92
AGCC^[[48]]^	2024	75.91	97.01	-	72.96	79.34	98.97	-	84.62
RCC^[[49]]^	2024	72.57	96.60	98.89	68.61	78.01	98.13	99.64	81.39
DNS^[[50]]^	ECCV24	77.27	-	-	**74.35**	84.21	-	-	*86.83*
MSCMNet^[[54]]^	2025	*78.53*	97.51	99.23	*74.20*	83.0	98.99	99.8	85.54
LAReViT^[[55]]^	2025	76.71	97.33	99.05	72.95	*84.22*	*99.02*	*99.85*	86.26
HIW(ours)		**85.05**	**97.67**	**99.67**	68.15	**87.50**	**99.32**	99.11	**87.75**

### Ablation studies and analyses

In this section, we conduct ablation studies and analyses to evaluate the effectiveness of each component in this paper proposed High-order Interaction and Wavelet Convolution Network (HIW). All experiments were conducted on the RegDB dataset under the same baseline in the IR-to-VIS mode. The wavelet convolution kernel size and shape loss weight are consistent. Results are shown in Table 3.

Adding each module alone increases the rank-1 metric by over 2.6% and the mAP metric by over 18%, proving each module’s effectiveness. When the wtDFM module and Shape dataset are added together, the rank-1 metric increases by 5.43%, and the mAP metric by 20.36%, this shows the complementarity of the wtDFM module and Shape dataset, both enhancing focus on shape features. Using all three modules at once boosts the rank-1 metric by 6.85% and the mAP metric by 23.34%.

**Table 3:** The impact of each component on HIW-Net. All experiments are conducted in the IR to VIS mode, with wavelet Convolution kernel sizes of $3 \times 3{\text{~}}$and $5 \times 5$, and shape loss and modality loss coefficients set to 0.1 and 0.9, respectively.

	Methods	RegDB
		rank-1/mAP
1	Baseline	87.47%/63.84%
2	Baseline + TPFI	90.8%/83.92%
3	+wtDFM	90.15%/82.00%
4	+shape	90.07%/83.03%
5	+TPFI + wtDFM	91.39%/84.01%
6	+TPFI + shape	91.17%/84.05%
7	+wtDFM + shape	92.97%/84.20%
8	+TPFI + wtDFM + shape	94.32%/87.18%

**Fig. 5:**
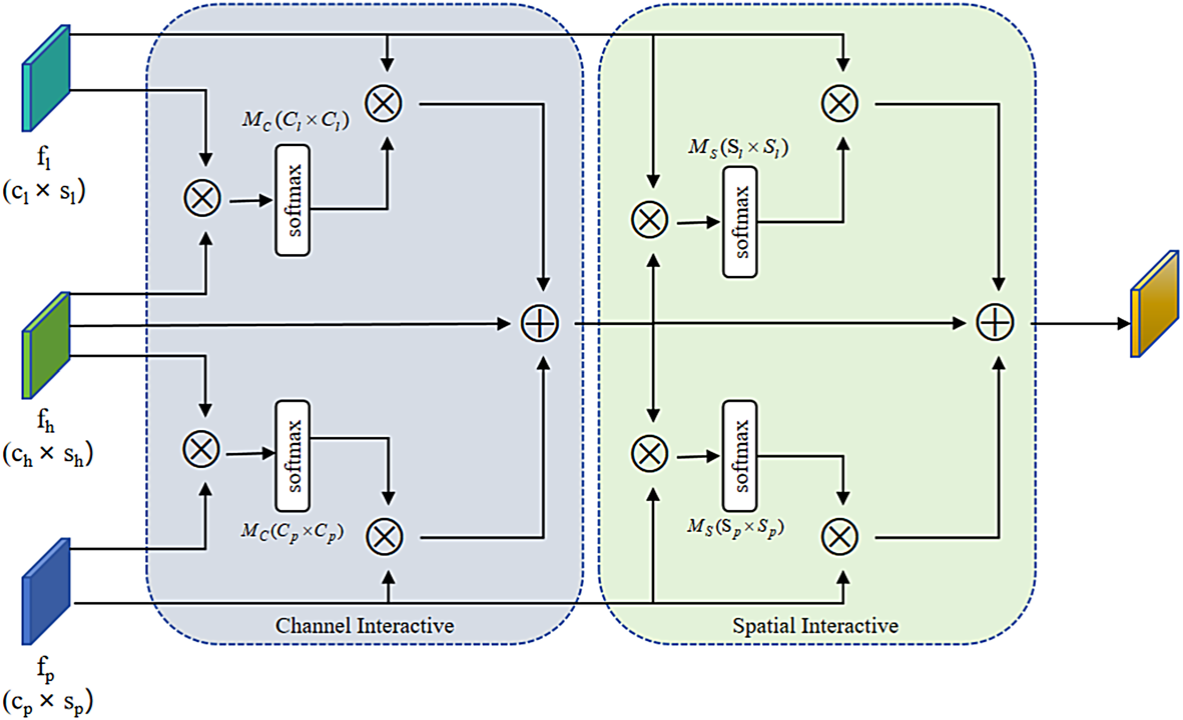
Third-order Primitive Feature Interaction.

**Fig. 6:**
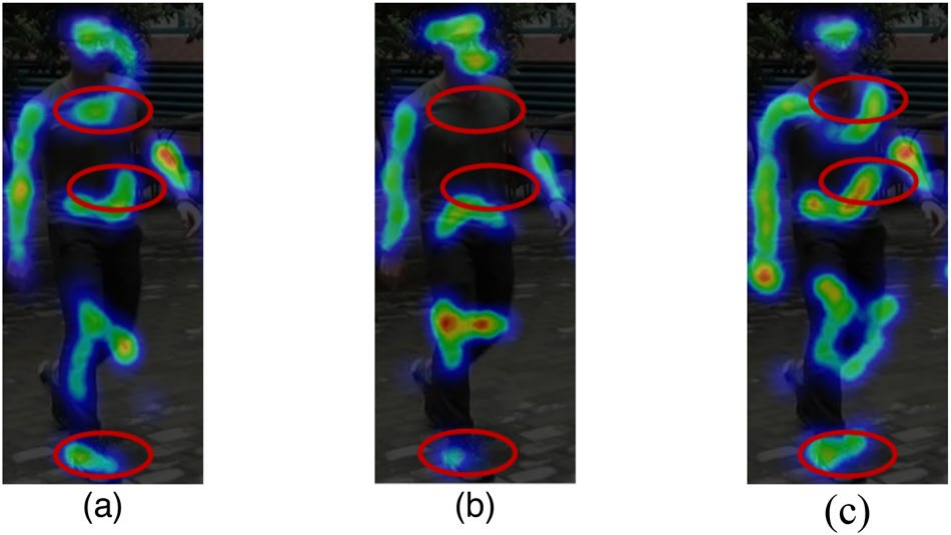
**a** represents the primitive feature map, **b** depicts the second-order feature interaction map, and **c** illustrates the third-order primitive feature interaction(TPFI) map.

**The effectiveness of third-order primitive feature interaction(TPFI): **As shown in Table 3. Adding the TPFI module to the baseline improves the rank-1 by 3.33% and the mAP by 20.38%. Visualizations in Fig. 5 show that features from general second-order interaction modules (Fig. 5.(b)) lose some chest, abdomen, and foot features compared to the primitive features (Fig. 5.(a)). However, Fig. 5.c, which uses the TPFI module to incorporate primitive features into deep network inputs, reduces such feature loss.(Fig .6)

**Fig. 7:**
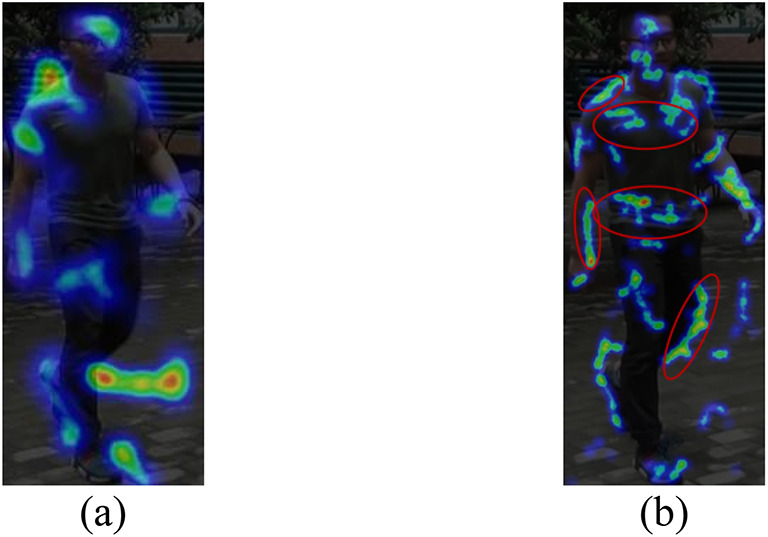
**a** shows the feature map of the module without wtDFM, while **b** depicts the feature map of the module with wtDFM.

**The effectiveness of diverse features mining for wavelet convolution(wtDFM)**As shown in Table 3, adding the wtDFM module to the baseline boosts the rank-1 by 2.68% and the mAP by 18.16%. Visualizations in Fig. 7.(b), which uses the wtDFM module, capture more features like the chest, abdomen, and legs, and focus more on the human silhouette than Fig. 7.(a), which doesn’t use the module.

To determine the optimal wavelet convolution kernel size for the wtDFM module, this section experiments with kernel sizes of $3 \times 3$ and $5 \times 5$, $3 \times 3$ and $7 \times 7$ and $5 \times 5$, as shown in Table 4. When the wavelet convolution kernel size is $3 \times 3$ and $5 \times 5$, the network achieves the best performance.

**Table 4:** The impact of wavelet Convolution kernel size on HIW-Net. All experiments set shape loss to 0.1 and modality loss to 0.9.

wtConv kernel size	RegDB(Rank-1)	
	VIS to IR	IR to VIS
$7 \times 7$ and $5 \times 5$	91.70	90.05
$3 \times 3$ and 7$\times 7$	92.33	92.33
$3 \times 3$ and $5 \times 5$	93.74	92.48

**Fig. 8:**
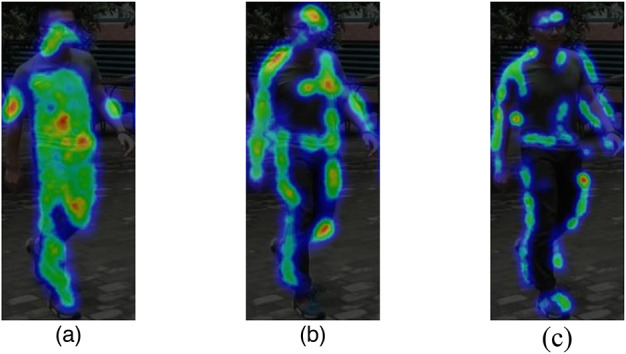
**a** represents the feature map of $a_{s}=0.1$, **b** represents the feature map of $a_{s}=0.2$, and **c** represents the feature map of $a_{s}=0.3$.

**The effectiveness of shape dateset**: As shown in Table 3, training on the baseline with the addition of the shape dataset increases the rank-1 accuracy by 2.60% and the mAP value by 19.19%. The weight coefficients of the model loss and shape loss are adjusted to explore the optimal combination, As shown in Table 5, when ${\alpha _s}=0.1$ and$~{\alpha _m}=0.9$, the network achieve the best performance. Figure 8 visualizes feature graphs of ${\alpha _s}=0.1$, ${\alpha _s}=0.2$, and ${\alpha _s}=0.3$, showing that as the weight coefficient of shape loss increases, the network places greater focus on shape features.

**Table 5:** The impact of weight coefficients ${\alpha _{s~}}$and $~{\alpha _m}$ on HIW-Net. All experiments used wavelet Convolution kernel size of $3 \times 3$ and $5 \times 5$.

${\alpha _s}$ and $~{\alpha _m}$	RegDB(Rank-1)	
	VIS to IR	IR to VIS
${\alpha _s}=0.3$,$~{\alpha _m}=0.7$	91.03	90.97
${\alpha _s}=0.2$,$~{\alpha _m}=0.8$	89.80	91.80
${\alpha _s}=0.1$,$~{\alpha _m}=0.9$	93.74	92.48

**Visualization Analyses**:

**Fig. 9:**
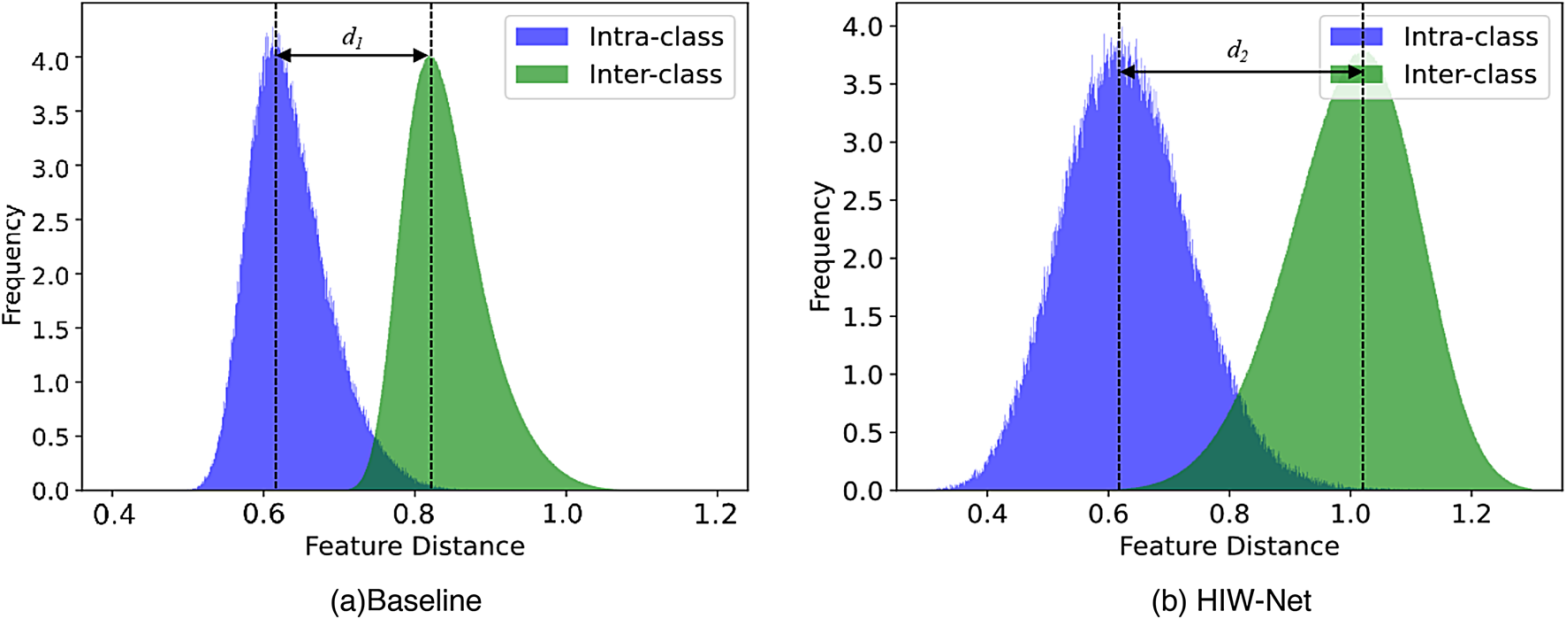
The feature distances of intra-and-inter classes visualization.

**Fig. 10:**
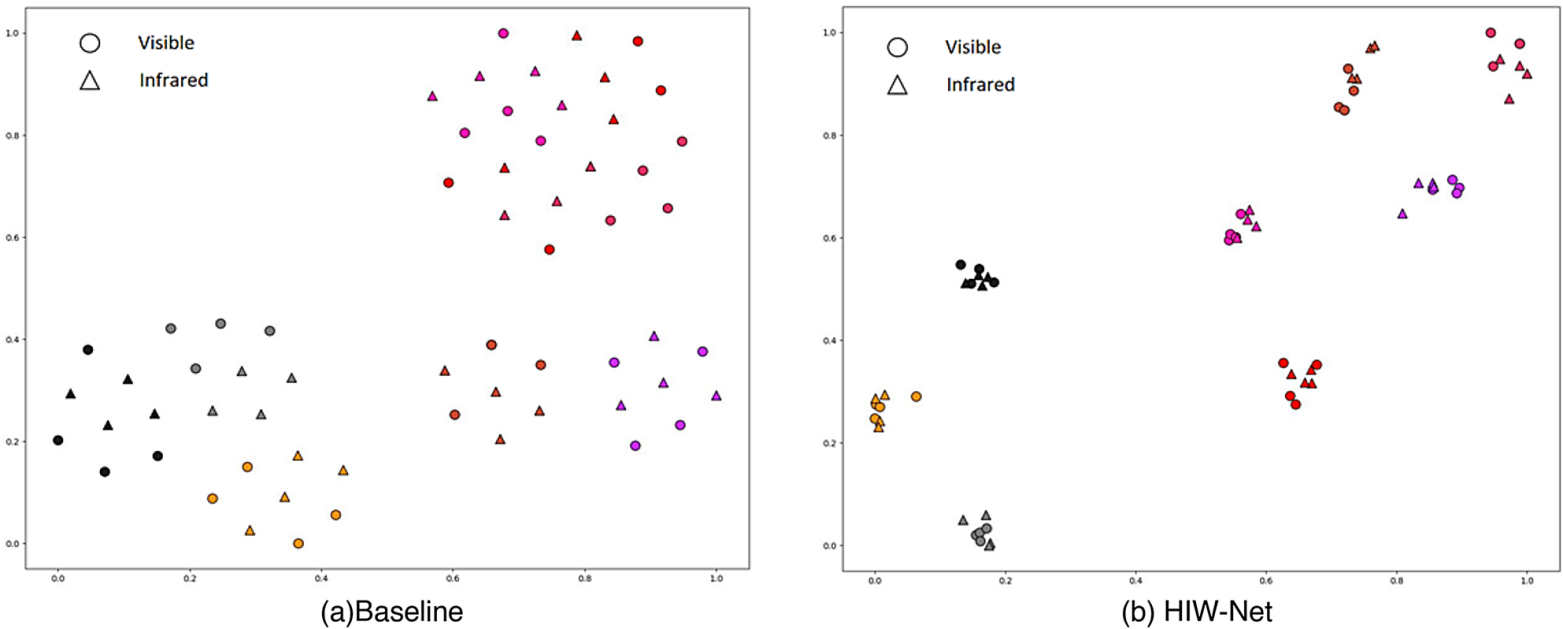
T-SNE visualization result of baseline and HIW-Net.

To investigate the effectiveness of HIW-Net, we visualize the inter class and intra class distances on the SYSU-MM01 dataset, as shown in Fig. 9. This indicates that HIW-Net achieves a larger gap between intra-class and inter-class distances, where ${d_1}<{d_2}$.Thus, HIW-Net can effectively reduce the modality discrepancy between the VIS and the IR images. As shown in Fig. 10, t-SNE visualization of the identity features learned by the model reveals that the baseline model’s projections for the same identity are scattered and hard to distinguish. In contrast, HIW-Net, leveraging TPFI and wtDFM, extracts more comprehensive and diverse features. This enables it to effectively distinguish and aggregate people features.

**Fig. 11:**
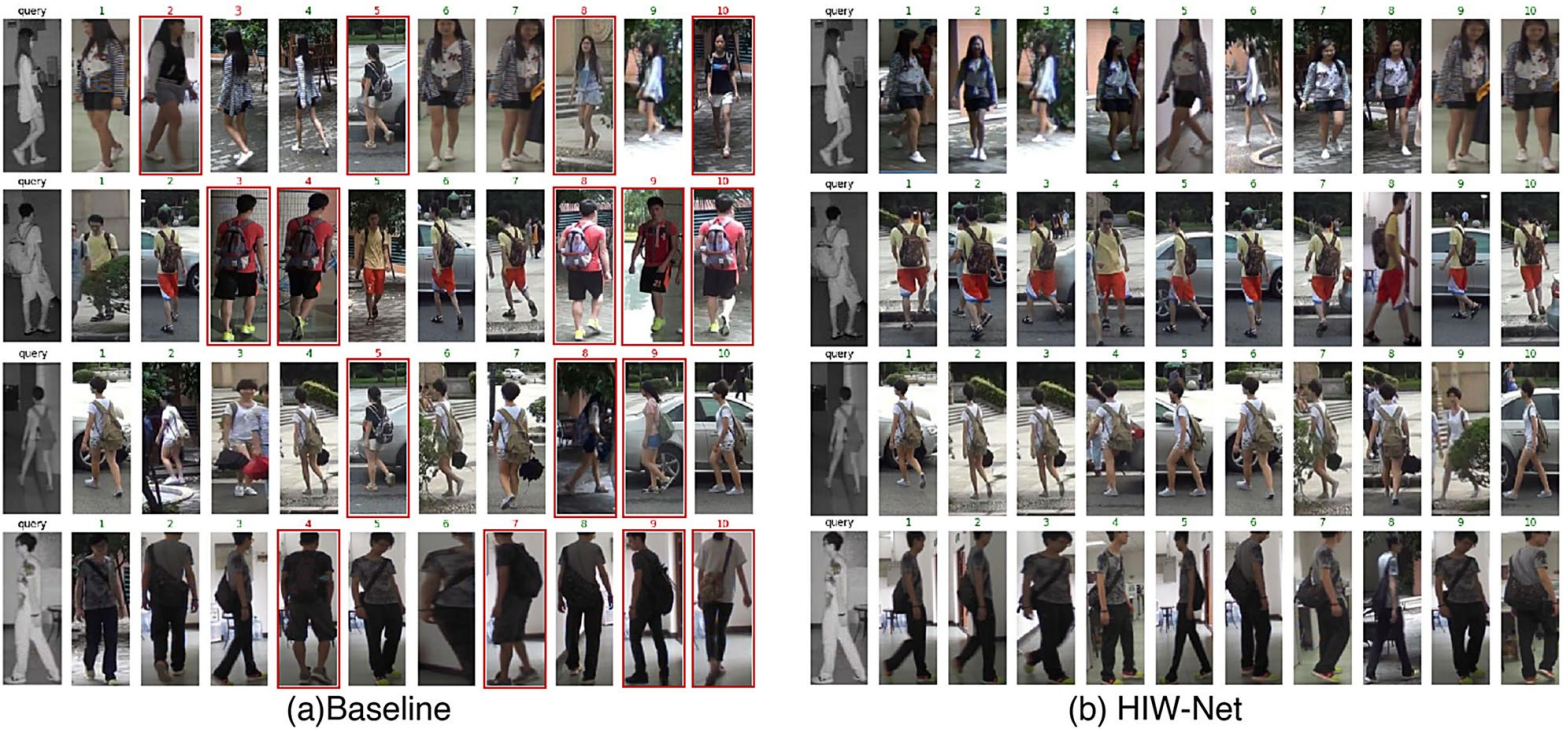
Some Rank-10 retrieval results obtained by the baseline and the proposed HIW-NET on SYSU-MM01 dataset.

To further show the effectiveness of HIW-Net, we also show some rank-10 retrieval results of HIW-Net on SYSU-MM01 dataset in Fig. 11, the red ones mean the incorrect matches. The results show that HIW-NET can achieve better person re-identification performance compared to the baseline.

## Conclusion

This paper addresses visible-infrared person re-identification by studying how to reduce feature loss during network feature extraction and more effectively mine features while reducing modality differences. The proposed HIW-Net, which consists of third-order primitive feature interaction (TPFI) and wavelet convolution diversity feature expansion (wtDFM), addresses these challenges. In addition, a shape loss weighting strategy is introduced to enhance the network’s attention to shape features. This paper also creates the RegDB_Shape dataset, manually annotating the low-quality RegDB dataset to generate shape maps of person. Extensive experiments on the SYSU and RegDB datasets demonstrate that the proposed HIW-Net outperforms existing methods.

## Data Availability

The SYSU-MM01 dataset and RegDB dataset used in the paper are publicly available and can be accessed through https://github.com/wuancong/SYSU-MM01 and https://github.com/WSR-001/HIW.The RegDB_Shape dataset is created based on RegDB and can be accessed through https://github.com/WSR-001/HIW.
